# The Effect of Innovation-Driven Strategy on Green Economic Development in China—An Empirical Study of Smart Cities

**DOI:** 10.3390/ijerph16091520

**Published:** 2019-04-29

**Authors:** Wenbin Cao, Ying Zhang, Peng Qian

**Affiliations:** Department of Management Science and Engineering, School of Business, Jiangnan University, 1800 Lihu Avenue, Wuxi 214122, China; aliazvv@163.com (Y.Z.); kanhuar@163.com (P.Q.)

**Keywords:** innovation-driven strategy, green development, smart city

## Abstract

Of all the countries presently industrializing, both going green and going smart ideas can be likened to green development, in that they are helping to address or identify new routes out of environment and development conflicts. Environmental pollution issues loom large for China, and the scale of its transformation makes its proposed solutions of widespread interest. China has begun to decouple some environmental pressure from economic growth, and an innovation-driven strategy is proposed to deal with the synergic development between the economy and environment. Considering both positive and negative effects of the innovation-driven strategy on green economic development, the aim of this paper is to find out the influence path and mechanism of policies and measures of the innovation strategy on green economic development, through the empirical analysis of panel data of 47 pilot smart cities in China from 2009 to 2017. The results show that the innovation-driven strategy has a positive effect on green development, while different innovation-driven indicators play different roles in the promotion of economic green development. Practically, this research provides a decision-making reference for sustainable development policy formulation in developing countries, especially in emerging economies.

## 1. Introduction

In recent decades, green economic growth has become a critical issue for most countries. For China, rapid economy development is mainly due to the low-cost advantage of the labor force and resource environment. The implementation of an innovation-driven development strategy is of long-term significance to reduce the consumption of resources and energy, and improve the ecological environment. This implies the requirements of making a transition from economic activities based on highly polluting energy sources to green economic activities based on technologies and consumptions with a lower environmental impact [1]. Technological change is necessary to enable a comprehensive response to environmental pollution without sacrificing economic growth. However, the problem is how we can make this transition possible. Schumpeter (1934) stated that economic development was driven by innovation [2]. The endogenous growth theory explains the influence of technological progress on the relationship between economic growth and the environment. The production process can be improved by increasing the substitution ability of polluted resources and replacing them with environment-friendly resources [3]. According to the endogenous economic growth model, Research and development (R&D) sectors use human capital and existing knowledge to create technological innovation [4]. In fact, economic theory highlights that the accumulation of R&D is of vital importance to economic growth. Nevertheless, economic growth based on the consumption of fossil fuels is closely related to environmental degradation from an environmental perspective. 

Innovation-driven strategy addressing the challenges of environmental pollution and achieving green development has acquired an important role in the economic literature in recent years [5,6]. Some literature has focused on examining the process of technological change [7]. Others study the optimal types of energy technologies to meet the demand for green development by taking into account economic and environmental parameters for the case of China, Greece, and Belgium [8]. Garrone and Grilli focused on public energy R&D, and empirically analyzed its relationship with carbon emissions per Gross domestic product (GDP). Evidence of causality links was obtained [9]. Their findings confirm that government R&D spending is not sufficient by itself to boost the innovation process. Public energy R&D has been successful in improving energy efficiency at the country level, but it has no correlation with the carbon factor and carbon intensity. Therefore, innovations do not necessarily have green attributes. They may promote or hinder urban eco-efficiency in accordance with multiple factors, such as industrial structures and regulatory policies. The effects of innovation strategy on green economic development are a combination of positive and negative effects.

Initially, the smart city notion was a spin off concept originating from the smart growth movement in the 1990s, which basically advocated planning strategies to address sprawl development and associated environmental externalities. However, the taking up of the concept of smart cities and, in general, of going smart could be signifying a departure from more traditional, greener narratives underpinning sustainable development. Questions can therefore be raised about the extent to which going green and going smart concepts overlap or better converge in their quest for sustainable development, or differ or diverge, leading to different directions or interpretations of sustainable development that might be prioritized. On this basis, can smart solutions assist policy in going beyond the aspirations of faux sustainability or “business as usual plus” to meet green development? [10].

Becoming the factory of the world has intensified the pressure on China’s environment. Industry is a major source of pollution, greenhouse gas emissions, and waste generation, which exert an increasingly adverse impact on human health and the environment. After the turn of the century, environmental pressure from rapid industrial expansion became a major policy issue. Air and water pollution and soil degradation have reached alarming levels. China’s energy consumption per unit of GDP is among the highest in the set of Organization for Economic Co-operation and Development (OECD) and BRICS (Brazil, Russia, India, South Africa and China) economies. It is also the world’s largest emitter of CO_2_ emissions since 2006, and is a source of enormous quantities of a range of pollutants. Industry is at the origin of many of these pressures. 

In the face of this problem, China has made great strides towards improving the environmental and resource productivity of its economy, and has made remarkable achievements in realizing the green development of the economy through innovation-driven strategy, in particular in pilot smart cities. China’s moves to introduce a green economy have attracted wide attention as a solution to severe problems of resource inefficiency and lack of resource productivity.

Considering both positive and negative effects of innovation-driven strategy on green economic development through empirical analysis of panel data of 47 pilot smart cities in China from 2009 to 2017, this paper aims to find out the influence path and mechanism of policies and measures of innovation strategy on green economic development to provide a decision-making reference for sustainable development policy formulation in developing countries, especially in emerging economies.

## 2. Theoretical Analysis and Research Hypothesis

Green economy has become one of the most fashionable terms in global environmental public policy discussions and forums, and it is selected as one of the organizing themes of the United Nations Rio+20 Conference in Brazil, June 2012. However, green economy as an effective mobilization tool for global environmental sustainability scholarship and practice remains unclear [11]. The thin green approach suggests that a green economy is a potentially useful corrective for industry and government to address the negative environmental impacts of industrial activity [12].

Scholars generally believe that there is a close relationship between economic growth and the ecological environment [13]. The purpose of a smart city is to create a greener, safer, and healthier urban development model [14]. However, for innovation-driven strategy as the core of smart city development, few literature has paid attention to the relationship between innovation-driven and green economy development of smart cities, and most existing studies are qualitative [15]. A small number of quantitative research is limited to the national level. Related achievements are difficult to guide the construction of smart cities. From the perspective of green economic development, the emissions of SO_2_, wastewater, and smoke and dust per unit of GDP are the main adverse indicators, and reflect the pollution situation in the process of regional economic growth. However, there has not been any systematic research to study the relationship between innovation-driven strategy and these three indicators. The innovation capability index refers to the innovation level of urban smart industries and R&D, including the development basis of innovation, innovation industrialization level, and R&D level. According to evaluation index system for smart cities, four main aspects are smart strategy, sector penetration, technical capability, and innovation capability. Innovation capability includes the level of social research and development, the level of business research and development, the level of science and technology service, the level of urban technology supply, the level of financial support, the level of technology application, the level of urban development, and the level of urban openness [16].Therefore, this paper thinks that innovation-driven strategy can be measured from four angles, namely, innovation input, innovation output, innovation vitality, and innovation subject. The hypothesis of its relationship with economic green development is put forward.

### 2.1. Innovation Input and Green Economic Development

Innovation input (*INI*) refers to the government’s investment in innovation activity through financial means Most scholars agree that government financial means play an important role in alleviating market failure and realizing optimal allocation of innovation resources [17], and that a government’s innovation investment and an enterprise’s innovation investment are correlated. Based on a U.S. sample, Scott found that government R&D investment could promote enterprise R&D investment [18]. Chinese enterprises are in a period of economic transition, and technological innovation is characterized by high investment, high risk, and long cycle return [19], which leads to insufficient investment in self-issued innovation. Therefore, efficient government management and investment can improve the innovation-driven level. Zhang et al. declared that the government could reduce SO_2_ emissions and smoke pollution in the process of urban economic growth by appropriate means of fiscal administration [20]. Xie and Saltzman suggested that government subsidies of environmental governance were conducive to reducing the discharge of wastewater per unit of GDP [21]. This research believes that an increase of innovation input is beneficial to improving and promoting green economic development. As a result, the following hypotheses are proposed in this study:

**H_1_**: *Innovation input is positively related to the green economic development of smart cities.*

**H_1a_**: *Innovation input is negatively correlated with SO_2_ emissions per unit of GDP in smart cities.*

**H_1b_**: *Innovation input is negatively correlated with the discharge of wastewater per unit of GDP in smart cities.*

**H_1c_**: *Innovation input is negatively correlated with the discharge of smoke and dust per unit of GDP in smart cities.*

### 2.2. Innovative Output and Green Economic Development

Innovation output (*INO*) refers to advanced patents and technology [22]. Grossman and Chen proposed that technological innovation had a positive effect on environmental quality improvement, and patents played an important role in it [23]. Chesbrough argued that a patent was the result of enterprise-specific strategy, which could affect the performance of enterprise innovation [24]. The increase of the number of patents has helped to boost the urban green total factor productivity. The transformation of patent achievements is an important measure to enhance the ability of urban innovation, that is, patents can be converted into science and technology directly applied to production practice through research, experiments, and other links. At the same time, the technological innovation of energy-saving and emissions reduction has the characteristics of path-dependency, which cannot only save the cost of production, but also protect the environment [25]. This research believes that the number of patent applications as are presentation of innovation output in smart cities can promote technology innovation of energy conservation and emissions reduction, and push forward green development. Accordingly, the following assumptions are made:

**H_2_**: *Innovation output is positively related to green economy development of smart cities.*

**H_2a_**: *Innovation output is negatively correlated with SO_2_ emissions per unit of GDP in smart cities.*

**H_2b_**: *Innovation output is negatively correlated with the discharge of wastewater per unit of GDP in smart cities.*

**H_2c_**: *Innovation output is negatively correlated with the discharge of smoke and dust per unit of GDP in smart cities.*

### 2.3. Innovative Subject and Green Economic Development

The innovation subject (*INS*) refers to innovative talents, which play a positive role in economic development and human progress [26]. Innovation talents are the driving force of innovation-driven development. Innovative talents not only have specific knowledge in a certain field, but also have a strong spirit of exploration and innovative thinking. In the process of accelerating urbanization, industrial clusters, scientific research institutes, universities, and other institutions in the region form an innovation network system, which can effectively reduce costs, form knowledge spillovers, and activate resources, thus creating a good innovation-driven atmosphere. Especially in the process of industrialization, the advantage of innovative talents is not only reflected in the research and development of new environmental protection technology, but also in the production practice from the top architecture design to the planning and implementation of sustainable development. This study believes that an increase of the number of innovative talents in smart cities can promote green economy development. As a result, the following assumptions are put forward:

**H_3_**: *Innovation subject is positively related to the green development of the smart city economy.*

**H_3a_**: *Innovation subject is negatively correlated with SO_2_ emissions per unit of GDP in smart cities.*

**H_3b_**: *Innovation subject is negatively correlated with the discharge of wastewater per unit of GDP in smart cities.*

**H_3c_**: *Innovation subject is negatively correlated with the discharge of smoke and dust per unit of GDP in smart cities.*

### 2.4. Innovation Vitality and Green Economy

Innovation vitality (*INV*) refers to the upgrading of industrial structure (reference). The change of industrial structure is manifested in the transformation of industrial structure elements, for example, from labor-intensive to capital-intensive, and then from capital-intensive to technology-intensive. Lahorgue and Cunha took developing regions as samples to study the upgrading of industrial structure, and found that the upgrading level could reflect the regional innovation level [27]. Xu and Feng proposed that the key point of industrial upgrading in China was in the innovation ability of enterprises, and the local market scale could be formed by rapid technological innovation [28]. Emerging countries can rapidly upgrade their industries by using new technologies to innovate. The tertiary industry is more “clean” than the first and the second industry, and the intrinsic richer sense of innovation can stimulate people’s environmental will and action. This study suggests that the promotion of innovation vitality reflected by the upgrading level of industrial structure can promote the green economy development of a smart city. Accordingly, the following assumptions are made:

**H_4_**: *Innovation vitality is positively related to the green economic development of smart cities.*

**H_4a_**: *Innovation vitality is negatively correlated with SO_2_ emissions per unit of GDP in smart cities.*

**H_4b_**: *Innovation vitality is negatively correlated with the discharge of wastewater per unit of GDP in smart cities.*

**H_4c_**: *Innovation vitality is negatively correlated with the discharge of smoke and dust per unit of GDP in smart cities.*

## 3. Data and Empirical Methodology

**Figure 1:**
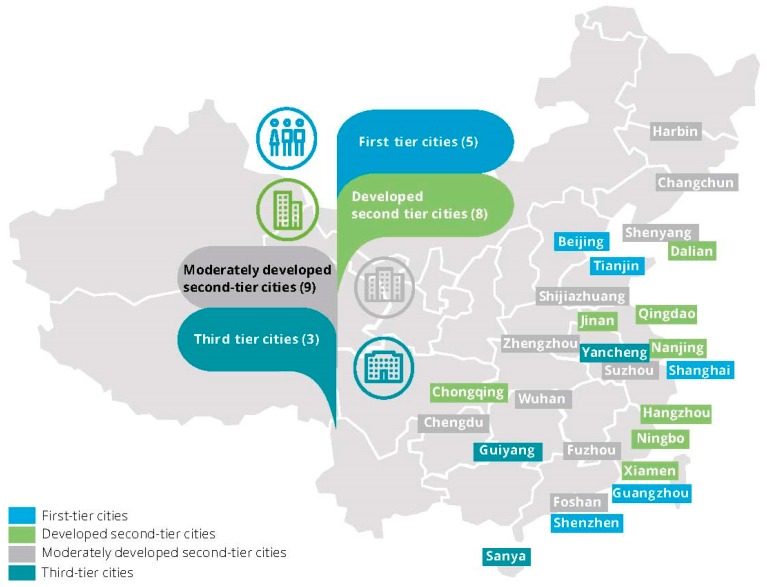
Distribution of smart city samples (source: zhihuichengshi.cn).

American international business machine co., Ltd (IBM) first proposed “smart city planning” to China in 2009.Hence, we chose data sources from 2009 to 2017. Judging from the pilot situation of smart cities in China, the Ministry of Housing and Construction began to apply for and approve smart cities in 2012. So far, it has issued three pilot lists of smart cities, the first of which was released in January 2013(see Figure 1). Smart cities were identified by the list of 50 smart cities in the 2017 “China Smart City Evaluation Model and basic Evaluation Index system” report. Because some variable data in Sanya, Dali, and Lhasa were missing, they were eliminated from the data pool. In total, 47 samples of smart cities were obtained.

### 3.1. Estimation Methodology

In order to reveal the relationship between innovation-driven strategy and green economy development in smart cities, the panel data model was constructed as follows:

(1) $$SO2_{ij}=\beta_{10}+\beta_{11}INI_{ij}+\beta_{12}INO_{ij}+\beta_{13}INS_{ij}+\beta_{14}INV_{ij}+\beta_{15}GDP_{ij}+\beta_{16}FDI_{ij}+\beta_{17}INF_{ij}+u_{10}$$

(2) $$WATER_{ij}=\beta_{20}+\beta_{21}INI_{ij}+\beta_{22}INO_{ij}+\beta_{23}INS_{ij}+\beta_{24}INV_{ij}+\beta_{25}GDP_{ij}+\beta_{26}FDI_{ij}+\beta_{27}INF_{ij}+u_{20}$$

(3) $$DUST_{ij}=\beta_{30}+\beta_{31}INI_{ij}+\beta_{32}INO_{ij}+\beta_{33}INS_{ij}+\beta_{34}INV_{ij}+\beta_{35}GDP_{ij}+\beta_{36}FDI_{ij}+\beta_{37}INF_{ij}+u_{30}$$

The above models investigated the effects of innovation-driven strategy on SO_2_ emissions (*SO*_2_), wastewater emission (*WATER*), and smoke-dust emissions (*DUST*) per unit of GDP in smart cities, respectively. Among them, *i* represents the city and *j* represents the year. *INI_ij_*, *INO_ij_*, *INS_ij_*, *INV_ij_*, *GDP_ij_*, *FDI_ij_*, and *INF_ij_* denote the innovation input, innovation output, innovation subject, innovation vitality, gross domestic product (*GDP*), total foreign direct investment (*FDI*), and infrastructure of city *i* in *j* year, respectively. *u* stands for random disturbance terms.

### 3.2. Dependent Variable

In this study, green economic development is measured by three inverse indicators, i.e., *SO*_2_, *WATER*, and *DUST*. The influence of innovation-driven strategy on green economic development can be investigated more comprehensively and intuitively through a comparison of different industrial pollution emissions. The data is from the “Environmental Statistical Yearbook” of every city.

### 3.3. Variables of Main Interest

The main explanatory variables of interest in this regression are *INI*, *INO*, *INS*, and *INV*. (1) Innovation input (*INI*). Judging from the present situation of china, the relative amount of expenditure on science and technology is insufficient [29]. Increasing the scale of our government’s investment in science and technology is an important way to improve our scientific research capability [30]. Therefore, the ratio of the government’s expenditure on science and technology to fiscal expenditure can reflect the situation of innovation investment; (2) innovation output (*INO*). At present, the patent is considered as the most important index of innovation output [31]. Therefore, this study selected the number of patent applications in each city as the measure of innovation output; (3) innovation subject (*INS*). A larger number of colleges and universities reflects a relatively larger number of students, which means a larger number of innovative subjects. Therefore, this study takes the proportion of college students to the local population as the main measure of innovation subject; (4) innovation vitality (*INV*). Developed countries tend to have more experience in innovation development and a stronger drive for innovation, and innovation-driven upgrading is usually increased in the proportion of third industries. Therefore, this study adopts the proportion of the third industry in GDP to represent innovation vitality. The data is from the “Science and Technology Statistical Yearbook”, “Demographic Yearbook”, and “Economic Statistics Yearbook” of every city.

### 3.4. Control Variables

Three control variables affect green economy development, including: (1) The economic level, denoted by local gross domestic product (*GDP*) of each city; (2) the open level, measured by the total amount of foreign direct investment (*FDI*); and (3) the infrastructure investment (*INF*), represented by road area per person at the end of the year. The data is from the “Economic Statistics Yearbook” of every city.

### 3.5. Descriptive Statistics

The calculation result shows that the values of the dependent variables are smaller. In order to avoid regression coefficients that are too small, the units of three dependent variables were processed to enlarge the values. At the same time, in order to enhance the comparability of the numerical values among the variables, the normalization method was used to calibrate the data, because the inconsistent units of the dependent variables may lead to the unsatisfactory results of the estimation. The value of the control variables was set as (0, 1) in order to enhance the comparability of the values among the variables, but it did not change its economic meaning. Variable descriptive statistics are shown in Table 1.

## 4. Empirical Results

### 4.1. Full Sample Regression Analyses

Based on the sample data of 47 cities, the relationship between innovation-driven strategy and green economic development was estimated by the individual fixed effect model. At the same time, in order to alleviate the heteroscedasticity in the regression process, the cross-section weighting method was adopted to further improve the estimation effect of the model. The measurement results are shown in Table 2.

First, using the model (1) to investigate the impact of innovation-driven strategy on SO_2_ emissions per unit of GDP in smart cities, it can be found that only *INS* and *INV* passed the test on the significance level of 1%. *INI* and *INO* failed to pass the test, that is, H_1a_ and H_2a_ are not supported, while H_3a_ and H_4a_ are supported. The results show that the higher the number of innovation subjects, the higher the level of tertiary industry development, and the lower the SO_2_ emissions per unit of GDP. The government’s financial investment in science and technology and the amount of patent applications have no effects on the SO_2_ emissions per unit of GDP.

Secondly, the model (2) was used to investigate the effect of innovation-driven strategy on the discharge of wastewater per unit of GDP. The results show that *INO*, *INS*, and *INV* have negative effects on the discharge of wastewater per unit of GDP. Therefore, H_1b_ is not supported, while H_2b_, H_3b_, and H_4b_ are supported. The results show that more patent applications, more colleges and universities, and a higher development level of the tertiary industry could effectively reduce wastewater emissions in economic development.

Finally, the model (3) was used to investigate the influence of innovation-driven strategy on smoke-dust emissions per unit of GDP in smart cities. The results show that *INI* and *INV* have negative effects on smoke-dust emissions per unit of GDP, while *INO* and *INS* have no effects on the smoke-dust emissions per unit of GDP. Hence, H_2c_ and H_3c_ are not supported, while H_1c_ and H_4c_ are supported. The results show that higher investment in science and technology in smart cities and higher levels of tertiary industry development are conducive to the reduction of smoke-dust emissions, while the number of patent applications and the proportion of universities and colleges in the total population have no effects on the reduction of smoke-dust emissions.

### 4.2. Sensitivity Analysis

It is important to note that there are four major cities (i.e., Beijing, Shanghai, Guangzhou, and Shenzhen) among all 47 samples of smart cities. The four major cities are far more powerful than other cities in both economic development and technological innovations, so testing them in the full samples could have an impact on the estimates. Therefore, this study eliminated the four sample cities, and then used the individual fixed effect model to estimate for sensitivity analysis.

The results of the sensitivity analysis are shown in Table 3. Compared with the results of the full-sample regression, first, *INS* and *INV* have negative effects on the SO_2_ emissions per unit of GDP after removing the four major cities based on the model (1), which is consistent with the regression result of the full-sample. Secondly, in the model (2), *INO* and *INV* passed the test, which shows that they have negative effects on the discharge of wastewater per unit of GDP. This is consistent with the result of the full sample regression. Although *INS* failed to pass the test, the coefficient symbol is still negative. This is basically consistent with the result of the full sample regression. Finally, in the model (3), *INI* and *INV* have significant negative effects on the smoke-dust emissions per unit of GDP, which is consistent with the regression result of the full sample. At the same time, the negative effect of *INO* on smoke-dust emissions per unit of GDP passed the test at a significance level of 10%, which is different from the results obtained by the full sample regression, but the influence is negative.

By comparing the results of the full sample test and the sensitivity analysis, it can be seen that the results are roughly the same, although there are differences in the results of individual variables, which may be due to the differences in the innovative capabilities and the level of green economic development between the four major cities and other cities. This may result in a small range of changes in the calculated results, but the estimated results are also robust. At the same time, the coefficients of all the passed-through variables are negative, which indicates that there is a real correlation between innovation-driven strategy and green economic development, because the index of green economic development is a reverse indicator. It shows that with the promotion of innovation-driven levels in smart cities, city economies can also develop in a green way.

## 5. Discussion

As a whole, innovation-driven strategy can promote green economy development, and different innovation-driven indicators have different influences on green development. Specifically, we obtained the following conclusions:(1) An increase of *INI* can significantly reduce the smoke emissions per unit of GDP, but the influence on SO_2_ and smoke emissions is not significant; (2) an increase of *INO* can reduce the discharge of wastewater per unit of GDP, but has no significant effect on the SO_2_ and smoke emissions; (3) an increase of INS can reduce SO_2_ emissions, but has no significant effect on wastewater and smoke emissions; and (4) an increase of *INV* has a significant inhibitory effect on the discharge of SO_2_, wastewater, and smoke-dust. Moreover, the sensitivity analysis results show that after excluding the samples from the four major cities, the measurement results are more robust, and the reliability of the empirical conclusion was confirmed again.

The influence of *INI*, *INO*, and *INS* on some indicators was not significant, which may due to the following reasons:

(1) China’s financial subsidies on innovation at present are overly concentrated on high-tech industries. China should be broadened to a wider range of sectors to boost innovation across the economy and maintain green growth. Economic green development needs sufficient financial support, and not all financial investment in science and technology is used for innovation R&D of economic green development. Hence, there may be unreasonable investment arrangements. In addition, there may be some problems in the process of fund utilization, such as supervision, imperfect incentives and evaluation mechanisms, and so on, so that the inhibition effect of innovation input on SO_2_ and smoke emissions is not significant.
(2) Although the number of innovation outputs, represented by patents, has increased dramatically in China, the quality of patents is relatively low, especially for the low level of innovation of anti-pollution technology. Thus, innovative output cannot effectively inhibit SO_2_ and waste water discharge. Moreover, the structure of talents is unreasonable and unbalanced, so it is difficult to give full play to the role of innovative talents in economic green development. Innovation vitality has a significant inhibitory effect on the three pollution indexes of smart cities in China, which indicates that the innovation vitality reflected by the upgrading of industrial structure in China has been effective in achieving environmental protection.

## 6. Conclusions

Based on the above analysis, in order to give full play to the effective role of innovation-driven strategy in green development of the economy, this study puts forward the following policy recommendations for developing countries or emerging economics: (1) Governments should use investment funds in science and technology and the supervision system reasonably. They should set up more targeted scientific and technological investment in pollution control and emissions reduction, and improve the scientific implementation of administrative means by improving the performance indicators of resources and the environment; (2) governments should establish a technical supply and management mechanisms of collaborative industry between the government and enterprises. The government should organize and guide regional scientific and technological forces to carry out collaborative innovation research and development, attach importance to the coordinated development of quantity and quality, and improve the proportion of patents for pollution control and emissions reduction and the conversion efficiency of real patents; (3) governments should pay more attention to the development of high-tech industries in the process of upgrading industrial structure in order to improve the efficiency of green production, promote the development of service industries to increase the proportion of clean industries, and make full use of the innovative vitality of the tertiary industry.

There are some limitations that affect the generalizability of the results of this study. Firstly, the data was gathered from China, and country-specific characteristics should be taken into account. However, China’s economic transformation and development model has a certain reference for developing countries. Thus, the results of this study can be utilized by developing countries or emerging economies. This research is cross-sectional in nature, which is a possible limitation of the research method employed. Further studies should investigate a wider scale of the drivers of green economy. Especially, the relationship between innovation and green economic growth requires further investigation. Innovation strategy could be studied together with other variables while it is possible that moderating or mediating effects could occur. Further research can also examine other effects of green development beyond the type of innovation investigated in this study.

## Figures and Tables

**Table 1:** Summary statistics for variables used for regressions.

Variable	Mean	Sd.	Min.	Max.
Dependent variables				
*INI*	0.13	0.15	0.00	1
*INO*	0.13	0.16	0.00	1
*INS*	0.42	0.26	0.02	1
*INV*	0.60	0.12	0.28	1
Independent variables				
*SO*_2_	0.23	0.23	0.00	1.61
*WATER*	0.28	0.23	0.03	1.57
*DUST*	1.08	1.35	0.00	8.88
Control variables				
*GDP*	0.20	0.17	0.00	1
*FDI*	0.10	0.13	0.00	1
*INF*	0.14	0.11	0.01	1

Notes: *INI*: Innovation input; *INO*: innovation output; *INS*: innovation subject; *INV*: innovation vitality; *SO*_2_: SO_2_ emissions; *WATER*: wastewater emission; *DUST*: smoke-dust emissions; *GDP*: gross domestic product; *FDI*: foreign direct investment; *INF*: infrastructure investment.

**Table 2:** Innovation-driven strategy and the impact on green economic development in smart cities.

Variables	Model 1		Model 2		Model 3	
Constant	1.19 ***	(21.62)	0.73 ***	(14.65)	2.76 ***	(10.27)
*INI*	−0.00	(−0.14)	0.01	(1.04)	−0.25 ***	(−2.66)
*INO*	0.02	(0.78)	−0.13 *	(−1.75)	−0.02	(−0.13)
*INS*	−0.63 ***	(−7.71)	−0.16 ***	(−2.61)	−0.41	(−1.27)
*INV*	−1.10 ***	(−10.56)	−0.41 ***	(−4.30)	−2.40 ***	(−5.10)
*GDP*	−0.06 **	(−2.30)	−0.42 ***	(−4.58)	−0.49 **	(2.07)
*FDI*	−0.11 *	(−1.90)	−0.19 ***	(−3.27)	−0.44	(−1.54)
*INF*	−0.33 ***	(−5.27)	−0.07	(−1.24)	−0.37	(−1.21)
Individual fixed effect	fixed		fixed		fixed	
R^2^	0.86		0.85		0.83	
Adjusted R^2^	0.84		0.83		0.81	
Prob. (F-statistic)	0.00		0.00		0.00	

Notes: ***, ** and * indicate the significance level of 1%, 5%, and 10%, respectively. The data in parentheses represents the *T*-value.

**Table 3:** Innovation-driven strategy and the impact on green economic development in smart cities after eliminating the four major cities.

Variables	Model 1		Model 2		Model 3	
Constant	1.16	(19.88)	−0.68	(15.41)	3.09	(9.74)
*INI*	−0.01	(−0.55)	0.00	(0.06)	−0.33	(−3.18)
*INO*	0.05	(0.67)	−0.31	(−2.61)	−0.48	(−1.91)
*INS*	−0.51	(−0.89)	−0.03	(−0.54)	−0.54	(−1.34)
*INV*	−1.00	(−9.70)	−0.29	(−3.30)	−2.80	(−4.75)
*GDP*	−0.48	(−4.20)	−1.06	(−7.37)	0.86	(1.72)
*FDI*	−0.02	(−0.30)	−0.00	(−0.05)	−0.69	(−1.98)
*INF*	−0.24	(−3.99)	0.03	(0.62)	−0.16	(−0.44)
Individual fixed effect	fixed		fixed		fixed	
R^2^	0.85		0.88		0.78	
Adjusted R^2^	0.83		0.86		0.75	
Prob(F-statistic)	0.00		0.00		0.00	

Notes: The data in parentheses represents the *T*-value.

## References

[1] Foxon T.J. (2011). A coevolutionary framework for analyzing a transition to a sustainable low carbon economy. Ecol. Econ..

[2] Schumpeter J.A. (1934). The Theory of Economic Development: An Inquiry into Profits, Capital, Credit, Interest, and the Business Cycle.

[3] Stokey N.L. (1998). Are there limits to growth?. Int. Econ. Rev..

[4] Romer P. (1986). Increasing returns and long-run growth. J. Polit. Econ..

[5] Costa Campi M.T., García-Quevedo J., Trujillo Baute E. (2015). Challenges for R&D and innovation in energy. Energy Policy.

[6] Sayegh S., Sanchez D.L., Caldeira K. (2017). Evaluating relative benefits of different types of R&D for clean energy technologies. Energy Policy.

[7] Popp D. (2010). Innovation and climate policy. Annu. Rev. Resour. Econ..

[8] Cheng R., Xu Z., Jones I. (2015). A multi-region optimization planning model for China’s power sector. Appl. Energy.

[9] Garrone P., Grilli L. (2010). Is there a relationship between public expenditures in energy R&D and carbon emissions per GDP? An empirical investigation. Energy Policy.

[10] Gazzola P., Del Campo A.G., Onyango V. (2019). Going green vs going smart for sustainable development: Quo vadis?. J. Clean. Prod..

[11] Ehresman T.G., Okereke C. (2015). Environmental justice and conceptions of the green economy. Int. Environ. Agreem. Politics Law Econ..

[12] Van der Ploega R., Withagen C. (2013). Green growth, green paradox and the global economic crisis. Environ. Innov. Soc. Transit..

[13] Grossman G.M., Krueger A.B. (1993). Environmental impacts of a North American free trade agreement. Mex. US Free Trade Agreem..

[14] Dameri R.P. (2011). Smart city and value creation. J. Urban Technol..

[15] Angelidou M. (2015). Smart cities: A conjuncture of four forces. Cities.

[16] Falco S.D., Studies E.P., Cooke P. (2019). Are smart cities global cities? A European perspective. Europ. Plan. Stud..

[17] Zhou H.T., Zhang Z.G. (2015). Research on the influence of government R&D funding on innovation investment and innovation performance of enterprises. J. Manag..

[18] Scott J. (1984). R&D, patents, and productivity. Firm Versus Industry Variability in R&D Intensity.

[19] Liu F., Yang Z. (2016). Institutional environment tax incentives and investment in enterprise innovation. Manag. Rev..

[20] Zhang Z.L., Zhou Y.H. (2012). Air pollution in China’s Economic growth and International comparison-taking sulfur dioxide as an example. Econ. Manag..

[21] Xie J., Saltzman S. (2000). Environmental policy analysis: An environmental computable general equilibrium approach for developing countries. J. Policy Model..

[22] Paroutis S., Bennett M., Heracleous L. (2014). A strategic view on smart city technology: The case of IBM Smarter Cities during a recession. Technol. Forecast. Soc. Chang..

[23] Chen S.Y. (2010). Energy conservation and emission reduction and win-win development of Chinese industry between 2009 and 2049. Econ. Stud..

[24] Chesbrough H.W. (2007). Open business models: How to thrive in the new innovation landscape. J. Prod. Innov. Manag..

[25] Aghion P. (2016). Carbon taxes, path dependency and directed technical change: Evidence from the auto industry. Soc. Sci. Electron. Publ..

[26] Wang P., Zhao J. (2011). Research on the Interactive relationship between industrial structure adjustment and regional innovation-based on interprovincial data of 2002–2008 in China. Res. Ind. Econ..

[27] Lahorgue M.A., Cunha N.D. (2004). Introduction of innovations in the industrial structure of a developing region: The case of the porto alegre technopole home brokers project. Int. J. Technol. Manag. Sustain. Dev..

[28] Xu K.N., Feng W. (2010). Endogenous industrial upgrading based on local market size: The third way to technological innovation. Ind. Econ. China.

[29] Bao J. (2010). Optimization analysis of china’s fiscal expenditure for science and technology. Sci. Manag. Res..

[30] Liu C.J., Liu S.Y. (2006). Government spending on science and technology: Scale and structure. J. Northeast Univ. Financ. Econ..

[31] Li J. (2013). Research on regional innovation agglomeration in China based on dynamic space panel Model. China Econ. Probl..

