# Corrrection: The Effect of Innovation-Driven Strategy on Green Economic Development in China—An Empirical Study of Smart Cities. Int. J. Environ. Res. Public Health 2019, 16(9), 1520

**DOI:** 10.3390/ijerph17103380

**Published:** 2020-05-12

**Authors:** Wenbin Cao, Ying Zhang, Peng Qian

**Affiliations:** Department of Management Science and Engineering, School of Business, Jiangnan University, 1800 Lihu Avenue, Wuxi 214122, China; aliazvv@163.com (Y.Z.); kanhuar@163.com (P.Q.)

The authors wish to add the following correction to their paper published in the International Journal of Environmental Research and Public Health [1]. 

The 16th reference, where the original expression is “Falco, S.D.; Studies, E.P.; Cooke, P. Are smart cities global cities? A European perspective. *Europ. Plan. Stud.* **2019**.”, should be changed to “Falco, S.D. Are smart cities global cities? A European perspective. *Europ. Plan. Stud.* **2019**, *27*, 759–783.”.

We apologize for any inconvenience caused to readers by this error.
